# Quality of facility-based maternal and newborn care around the time of childbirth during the COVID-19 pandemic: Marzia Lazzerini

**DOI:** 10.1093/eurpub/ckac129.189

**Published:** 2022-10-25

**Authors:** M Lazzerini, B Covi, I Mariani, Z Drglin, M Arendt, I Hersoug Nedberg, H Elden, R Costa, D Drandic, E Pessa Valente

**Affiliations:** 1 WHO Collaborating Centre, Institute for Maternal and Child Health IRCCS, Trieste, Italy; 2 National Institute of Public Health, Ljubljana, Slovenia; 3 Beruffsverband vun de Laktatiounsberoderinnen, Luxemburg, Luxembourg; 4 Department of Community Medicine, UiT The Arctic University of Norway, Tromso, Norway; 5 Institute of Health and Care Sciences, Sahlgrenska Academy, University of Gothenburg, Gothenburg, Sweden; 6 Department of Obstetrics and Gynecology, Sahlgrenska University Hospital, Gothenburg, Sweden; 7 EPIUnit, University of Porto, Porto, Portugal; 8 Hei-Lab, Lusofona University, Porto, Portugal; 9 IRT, Porto, Portugal; 10 Roda-Parents in Action, Zagreb, Croatia

## Abstract

**Background:**

Multi-country studies assessing the quality of maternal and newborn care (QMNC) during the COVID19 pandemic, as defined by WHO Standards, are lacking.

**Methods:**

Women who gave birth in 12 countries of the WHO European Region from March 1, 2020 - March 15, 2021 answered an online questionnaire, including 40 WHO Standard-based Quality Measures.

**Results:**

21,027 mothers were included in the analysis. Among those who experienced labour (N = 18,063), 41.8% (26.1%- 63.5%) experienced difficulties in accessing antenatal care, 62% (12.6%-99.0%) were not allowed a companion of choice, 31.1% (16.5%-56.9%) received inadequate breastfeeding support, 34.4% (5.2%-64.8%) reported that health workers were not always using protective personal equipment, and 31.8% (17.8%-53.1%) rated the health workers’ number as “insufficient”. Episiotomy was performed in 20.1% (6.1%-66.0%) of spontaneous vaginal births and fundal pressure applied in 41.2% (11.5% -100%) of instrumental vaginal births. In addition, 23.9% women felt they were not treated with dignity (12.8%-59.8%), 12.5% (7.0%-23.4%) suffered abuse, and 2.4% (0.1%-26.2%) made informal payments. Most findings were significantly worse among women with prelabour caesarean birth (N = 2,964). Multivariate analyses confirmed significant differences among countries, with Croatia, Romania, Serbia showing significantly lower QMNC Indexes and Luxemburg showing a significantly higher QMNC Index than the total sample. Younger women and those with operative births also reported significantly lower QMNC Indexes.

**Conclusions:**

Mothers reports revealed large inequities in QMNC across countries of the WHO European Region. Quality improvement initiatives to reduce these inequities and promote evidence-based, patient-centred respectful care for all mothers and newborns during the COVID-19 pandemic and beyond are urgently needed.

Funding: The study was financially supported by the Institute for Maternal and Child Health IRCCS Burlo Garofolo, Trieste, Italy.

